# Reliability of retinal vessel calibre measurements using a retinal oximeter

**DOI:** 10.1186/s12886-015-0174-0

**Published:** 2015-12-24

**Authors:** Rebekka Heitmar, Angelos A. Kalitzeos

**Affiliations:** Aston University, School of Life and Health Sciences, Aston Triangle, Birmingham, B4 7ET UK

## Abstract

**Background:**

Summarised retinal vessel diameters are linked to systemic vascular pathology. Monochromatic images provide best contrast to measure vessel calibres. However, when obtaining images with a dual wavelength oximeter the red-free image can be extracted as the green channel information only which in turn will reduce the number of photographs taken at a given time. This will reduce patient exposure to the camera flash and could provide sufficient quality images to reliably measure vessel calibres.

**Methods:**

We obtained retinal images of one eye of 45 healthy participants. Central retinal arteriolar and central retinal venular equivalents (CRAE and CRVE, respectively) were measured using semi-automated software from two monochromatic images: one taken with a red-free filter and one extracted from the green channel of a dual wavelength oximetry image.

**Results:**

Participants were aged between 21 and 62 years, all were normotensive (SBP: 115 (12) mmHg; DBP: 72 (10) mmHg) and had normal intra-ocular pressures (12 (3) mmHg). Bland-Altman analysis revealed good agreement of CRAE and CRVE as obtained from both images (mean bias CRAE = 0.88; CRVE = 2.82).

**Conclusions:**

Summarised retinal vessel calibre measurements obtained from oximetry images are in good agreement to those obtained using red-free photographs.

## Background

Retinal vascular calibres can be measured non-invasively using retinal photographs. In the last 10–15 years a standardised approach to measure and calculate summarised retinal vessel calibres has evolved [1, 2]. Thereby the summarised arteriolar and venular calibres are referred to as central retinal arteriolar equivalent (CRAE) and central retinal venular equivalent (CRVE) from which the arterio-venous ratio (AVR) can be calculated. While the AVR is a poor marker of vascular changes at the retinal level [3, 4], CRAE and CRVE have been shown more clinically useful. In hypertensive patients for example, a decreasing CRAE over time is associated with increased arterial stiffness (in early-stage hypertension) and has shown potential to be predictive in regards to cardiovascular mortality and morbidity. This provides data on the potential of CRAE to become a useful marker for risk stratification in hypertensive (HT) patients [5]. A systematic review by Ding J and colleagues [6] evaluating data of 10229 participants revealed an association of decreased CRAE and increased CRVE with an increased risk of HT. Larger retinal venular calibres are also predictive of stroke [7]. Furthermore, a decrease in CRAE and an increase in CRVE over time are predictive of microvascular outcome in type 1 Diabetes Mellitus (DM) [8].

Recently, there has been an increasing amount of ocular imaging devices available. Each device is developed to assess one or more markers. However, when measuring multiple parameters it is not practical to move participants constantly between instruments. Another limiting factor is acquisition and exposure time which can lead to non-compliance and increased drop-out rates of study participants.

Retinal vessel calibres are not only important to assess in regards to cardiovascular risk and microvascular changes at the retinal level but form an integral part of the analysis for retinal oximetry as it has been shown that the oxygen saturation is depending on vessel diameter [9]. Blondal and colleagues [10] have shown that diameter measurements obtained with their oximeter show good repeatability. While a given marker can be measured by using different techniques, this does not necessarily mean that these techniques can be used interchangeably (i.e. have comparable results), owing to their inherent differences. Here, we set out to explore two such markers – CRAE and CRVE – obtained with two different techniques: one using a red-free filter and the other one using a dual wavelength filter used for taking retinal oximetry images and extracting only the information from the green channel. CRAE and CRVE are more commonly used rather than using individual vessel calibres when examining their associations with vascular pathology. Hence, the focus of this study was not on individual vessel calibre agreement but on summarised vessel calibre agreement when using the two aforementioned acquisition techniques.

## Methods

### Subjects

The study followed the guidelines of the Declaration of Helsinki and was approved by the Aston Optometry and Audiology Research Ethics Committee. All participants gave written informed consent prior to inclusion in the study. We included 45 arbitrarily selected eyes (25 left and 20 right eyes) of 45 healthy participants (age range: 21 – 62 years). Twelve subjects were of South Asian (SA) background and thirty-three of Caucasian (CA) origin. All participants initially underwent non-contact tonometry (Pulsair, Keeler, UK) followed by pupil dilation using one drop of Tropicamide 1 % (Bausch & Lomb, UK). After a minimum of 15–20 min acclimatisation in a temperature controlled room (21 ± 2 °C) systemic blood pressure and heart rate was measured using a digital sphygmomanometer (UA-767, A&D Instruments Ltd., UK).

### Image acquisition and analysis

**Fig. 1:**
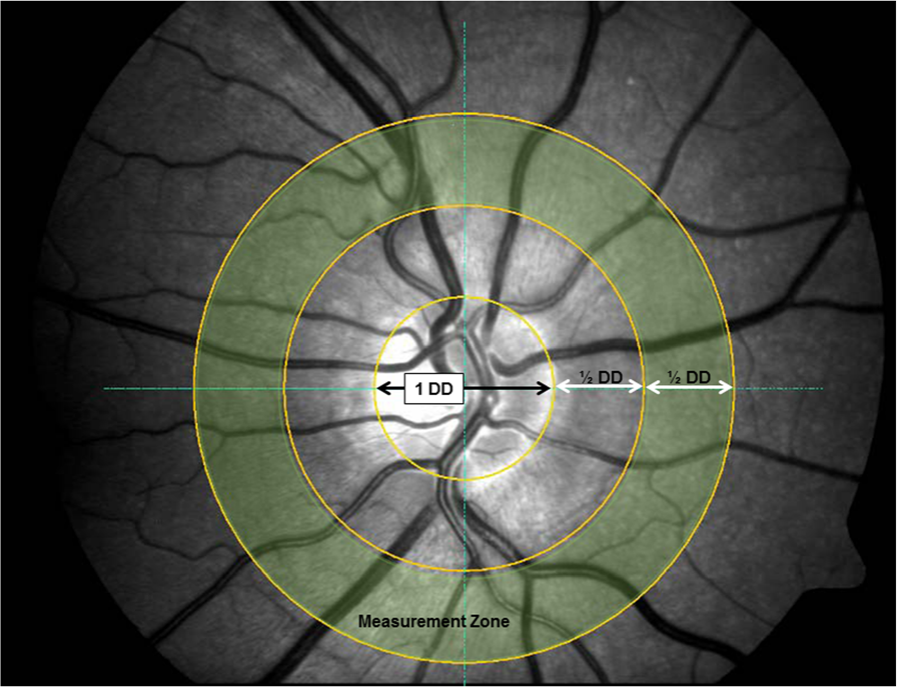
Sample retinal image with optic nerve head centred and measurement zone from which retinal vessel calibres were obtained

Following full pupil dilation, first the monochromatic (red-free) and after a resting period of a minimum of 5 min the oximetry (dual-wavelength) retinal photographs were obtained using a Zeiss FF450+ fundus camera (Zeiss, Germany and Imedos Systems, Germany) with the optic nerve head (ONH) centred and the camera field angle set to 30° (see sample image and measurement area in Fig. 1). One image obtained with the dual wavelength filter (specifications: 548 ± 10 nm (full width at half maximum) and 610 ± 10 nm) and one monochromatic image were used to measure the CRAE and CRVE of each image; more detail on the spectral transmission of the dual wavelength filter used for retinal oximetry can be found elsewhere [9]. Room illumination was switched off during imaging.

In brief, retinal vessel diameters were measured using a semi-automated piece of software (VesselMap 2, Imedos, Germany) by a single observer (OS). Following image selection, two concentric rings with ½ Disc Diameter (DD) and 1 DD were placed distant from the ONH. The same vessels were assessed in both images. The six largest retinal arteries and veins passing through the ring segment (see Fig. 1) were included in the analysis as recommended by Knudtson and colleagues [2]. The formulae used to derive CRAE and CRVE after three iterations are provided in Eqs. 1, 2 (Parr-Hubbard method)^1^ below:

1 $$ {\mathrm{W}}_{\mathrm{artery}}=\sqrt{0.87\ {W}_1^2+1.01\ {W}_2^2-0.22{W}_1{W}_2-10.76} $$

2 $$ {\mathrm{W}}_{\mathrm{vein}}=\sqrt{0.72{W}_1^2+0.91{W}_2^2+450.05} $$

where W_artery_ denotes an estimated arteriolar trunk width from the pair of the narrower and wider retinal arteriolar branches, W_1_ and W_2_, respectively. Similarly, W_vein_ denotes an estimated venular trunk width from the pair of the narrower and wider retinal venular branches, W_1_ and W_2_, respectively. After the first formula iteration the six blood vessels are reduced to three estimated diameters. The narrower and wider vessels of these three are again combined into a new trunk estimate, whereas the remaining vessel is carried over to the next (and final) formula iteration to derive the CRAE or CRVE. From these two parameters AVR is then calculated as follows: AVR = CRAE/ CRVE.

### Statistical analysis

Bland-Altman analysis was performed to evaluate the agreement [11, 12] between the calibre parameters obtained (CRAE, CRVE and AVR) from the two images. For ease of visualisation we have included a scatterplot for CRAE and CRVE values for the two sets of images. All data were analysed using GraphPad Prism (Version 6.05, GraphPad Software, California USA).

## Results

All forty-five participants (mean (SD) age: 34 (10) yrs) were normotensive (SBP: 115 (12) mmHg; DBP: 72 (10) mmHg) and had IOPs within a normal range (12 (3) mmHg).

**Fig. 2:**
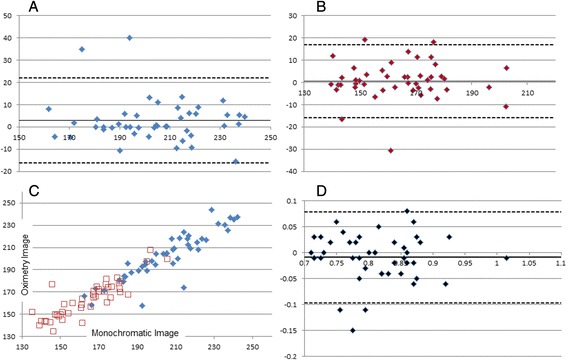
Bland Altman plots for (**a**) CRVE; (**b**) CRAE and (**d**) AVR. The solid horizontal line denotes the mean difference between the two summarised vessel calibres as obtained from the red-free and the oximetry image. The two dotted lines show the ±1.96 standard deviations of the difference (denoting the upper and lower limits of agreement). Scatter plot showing the correlation of (**c**) CRAE (*red*) and CRVE (*blue*) between the two images

Results from the Bland-Altman analysis showed good agreement (mean bias of CRAE, CRVE and AVR is approaching zero) between the two images under comparison. Detailed results including the mean bias (or sometimes referred to as the mean difference), upper and lower limits of agreement can be found in Table 1 and as a graphical representation in the four plots in Fig. 2.

**Table 1:** Summarised retinal diameters and results from the Band-Altman analysis

	Monochromatic Image	Oximetry Image	Bias (mean difference)	Lower limit of agreement	Upper limit of agreement
	*Mean (SD)*	*Mean (SD)*	*Mean (SD)*		
CRAE (μm)	164.36 (16.63)	163.47 (16.48)	0.65 (8.36)	−15.75	17.04
CRVE (μm)	205.40 (20.56)	202.57 (21.46)	2.82 (9.75)	−16.29	21.93
AVR	0.81 (0.07)	0.81 (0.07)	−0.01 (0.04)	−0.09	0.08

*CRAE* central Retinal Arteriolar Equivalent, *CRVE* Central Retinal Venular Equivalent, *AVR* Arterio-Venous Ratio

## Discussion

The Bland-Altman analysis showed that the vessel parameters obtained from both images are in good agreement. When observing the plots it is also apparent that the larger arteriolar and venular equivalents show less spread. This is not surprising, as larger arteries and veins can be measured with higher accuracy due to their size and contrast profile.

Retinal vascular calibres can be used as standalone measurements to evaluate the retinal microcirculation, associations with systemic and ocular vascular disease such as glaucoma [13], diabetes [8, 14, 15], hypertension [5, 16], stroke [17] and cardiovascular abnormalities [18, 19]. Since the possibility of obtaining more “dynamic” and “metabolic” data of the ocular circulation through the introduction of retinal vessel analyser (RVA) [20] and retinal oximetry [21], vessel calibres are more commonly used in conjunction with these more novel parameters as well as forming the foundation for correcting influences of vessel size [8]. However, when calculating parameters such as retinal vessel oxygen saturation and blood flow (BF), it is paramount to have accurate vessel calibre measurements as they form the basis of the calculation for some (i.e. BF) as well as complementing other parameters for a more complete insight into the retinal microcirculation.

While some oximeters integrate the vessel calibre measurements into their saturation calculation, they don’t provide automatic output of summarised vessel calibres. These can however be calculated by extracting the green channel information (i.e. red-free image) of the image obtained as in our example. The hitherto additional parameter acquired can be used to compare to previously obtained vessel calibres as well as in conjunction with other metabolic or structural markers measured as part of an examination. The use of the oximeter output rather than an additional monochromatic image using a standard green filter also means less exposure for the patient and shorter acquisition time for the clinician.

## Conclusion

Retinal photographs obtained by a retinal oximeter can be used to measure and calculate summarised retinal vessel calibres in good agreement with those obtained using a standard red-free filter. This enables the extraction of another important marker for the assessment of retinal microvasculature in addition to retinal vessel oxygen saturation values without the need of taking additional monochromatic images.
